# Foreign Summary

**Published:** 1839-03-30

**Authors:** 


### FOREIGN SUMMARY.

*Rupture of the Urinary Bladder from external injury.* By Wm. Lawrence, F. R. S., &c.—James Taplin, 35 years of age, was admitted into the hospital on Monday July 2d, in consequence of an accident which he had met with the day before. He was in a cabriolet, which had been overturned, and the vehicle had fallen on his abdomen. He had not made water for some time, and therefore concluded that his bladder must have been full at the time of the accident, since which he had voided no urine. The abdomen was painful and tense in a slight degree, towards the lower part; above, it was tolerably soft and compressible. Pulse quick and feeble; respiration short and hurried; tongue dry; countenance anxious. There was occasional vomiting. A catheter was introduced, but no urine flowed; when the instrument was withdrawn, its end was slightly smeared with blood. A few leeches were applied to the hypogastric region, and followed by fomentation.

One grain of opium every four or six hours.

July 3d.—No urine has been voided, either naturally or by the catheter, which has been again introduced. The symptoms are worse, though without acute suffering. Pulse 140.

4th.—Rejection, by vomiting, of a black fluid, giving the tongue a similar colour, quite different from its dark appearance in typhus. Pulse sinking; the cerebral functions not disturbed. He died in the evening.

6th.—*Examination of the body.*—The bladder presented, on the posterior aspect of its fundus, a rupture more than an inch in length, through all the tunics. It was a tolerably clean division, with the edges a little ecchymosed. There was general peritonitis, of which the appearances were most strongly marked in the neighbourhood of the bladder. The abdomen contained a turbid fluid, of tawny colour, to the amount of four or five pints; it had no urinous smell. The mucous membrane of the air-passages, and that of the stomach, were congested. The other organs were healthy.

The escape of urine into the abdomen, and its rapid diffusion over the cavity, excite general peritonitis, attended with serious depression of the vital powers; and death ensues in short time, under circumstances which seem to render our efforts unvailing and hopeless. In the present case, the bladder had been full at the time of the accident; twenty-four hours had elapsed without any attempt at relief. The viscus was then found to be empty, so that three or four pints of urine must probably have passed into the abdomen. The catheter should be introduced as early as possible in a case of ruptured bladder; and it would probably be advisable to leave it in permanently, so that the urine might flow off externally. The violently irritating effects of this fluid on surfaces with which it comes into accidental contact, hardly allow us to hope that the peritonitis can be controlled in these cases, more especially as the state of the circulation and that of the nervous system entirely preclude active depletion. The wound in the bladder cannot be expected to unite, as its sides must be moistened with urine. If the patient's life could be prolonged, the breach in the bladder might possibly be closed by the adhesion of the neighbouring viscera. No instance, however, has come to my knowledge of recovery from this injury.

It appeared to me, in the present case, that the administration of opium had an advantageous effect in alleviating suffering.

Two cases of destructive, I might probably say malignant disease, have lately occurred in the hospital, and have terminated fatally within the last few days. Having the opportunity of shewing you the morbid parts, I will at the same time mention shortly the particulars of these cases.—*Lond. Med. Gaz.*

---

*On Disengagement of a Gaseous Fluid in the Circulation, as a Cause of Sudden Death.* By Dr. Ollivier.—After noticing the common causes of sudden death, such as cerebral and spinal apoplexy, pulmonary apoplexy, and emphysema, rupture of the heart or of the large vessels, Dr. Ollivier asserts that many cases occur in which an examination after death demonstrates no organic lesion, and consequently that the cause of death is by no means apparent or satisfactorily accounted for. The author's object in the present paper is to prove the possibility of a spontaneous development in the blood during life, of a gaseous fluid, which produces instantaneous death by its accumulation in the right cavities of the heart; either as an obstacle purely mechanical to the circulation, if the gas is analogous to atmospheric air; or as a deleterious agent, if, as may be supposed, it is carbonic acid gas which is disengaged. The following is a case which is supposed to support this view.

A young woman died suddenly, at the moment she was endeavouring to get up. Upon examination nothing could be found to account for death, but a considerable dilatation of the right cavities of the heart by a gaseous fluid. She had been suffering for some time from debility, which was sensibly increased, without any apparent cause, upon the day of her death.—*Brit. and For. Med. Rev., from Revue Médicale.*

---

*On the Removal of the Bones of the Face.* By Professor J. F. Dieffenbach.—The attention which modern surgeons have given to remedy or remove the diseases which attack the face, has induced Dr. Dieffenbach to give his experience on the subject, in the form of a report of a number of cases in which he had successfully removed portions or the whole of the upper and lower jaw.

The superior maxilla was first removed by Professor Lizars, of Edinburgh. Since his time numerous surgeons, both in Great Britain and on the continent, have performed this operation. In a paper by Mr. Liston, published in the 20th volume of the Medico-Chirurgical Transactions, on this subject, an account is given of those diseases which affect the upper and lower maxilla; and those are distinguished in which an operation may be ventured upon with hopes of success. The diseases are the following: 1. Parulis, or spina ventosa; swellings of an acute or chronic nature, from which there is usually a purulent secretion. 2. Epulis; a solid growth from, and of the consistence of gum. 3. A fungous vascular growth from the apex of the fangs of teeth, which increases gradually, bleeds easily, and beneath which the osseous tissue becomes changed into a soft lardaceous brain-like matter. 4. Osteo sarcoma; a soft, ragged, foul, ulcerated, fungous growth, commencing usually amongst the bones, which it gradually displaces; sometimes bleeding, and always malignant. 5. Tumours of erectile tissue, usually occupying the antrum. 6. Simple fibrous tumours, having a botryoidal form, and slow in growth, attaining a great size, and not malignant.

The parulis and epulis are usually slight affections, involving only small portions of the alveolar process, and which may be easily removed. The fibrous tumours may always be safely removed: they are slow in growth, and not of a malignant character. The other diseases should only be interfered with at an early period, when the neighbouring glands are not affected, and when the whole disease, together with the surrounding healthy parts, may be removed.

Of the cases reported by Dieffenbach, twelve involved only portions of the jaw, and were usually of the nature of the parulis or epulis. Three cases, in which the whole of one of the superior maxillary bones was removed, would appear, from the slowness of their growth, and the size they had attained, to have been of the nature of the fibrous or botryoidal tumours of Mr. Liston. One case of the lardaceous or brain-like affection of the bone was submitted to five different operations before the disease was extirpated. M. Dieffenbach calls one case Fungous malonodes, and which he successfully treated by removal of the superior maxillary bone. A fibrous tumour of the lower jaw was also removed successfully, together with a portion of the bone.

All the cases reported by Dieffenbach had a favourable termination.

[After the first report of fifteen cases of removal of the upper maxillary bone, and of which eleven proved fatal, this operation was unfavourably thought of. But if we keep in mind the rules of diagnosis laid down by Mr. Liston, and consider the termination of the various cases which have been operated on, we may consider this as one of the most successful operations in surgery. Mr. Liston has removed the superior maxilla seven times, with only one case of failure. Dr. Dieffenbach has had five operations, and no failure. Gensoul has removed the superior maxilla four times, with one case of failure; and then the affection was of a malignant nature. Regnoli had one successful case, and one failure. Of the eleven cases operated on by Messrs. Lizars, Syme, Robert, Scott, Earle, Guthrie, and Hetling, only one case was completely successful; but no regard was paid to the malignant or simple nature of the disease.

In the performing of the operation of removing the upper jaw, Dr. Dieffenbach insists much on the necessity of dividing the skin as much as possible in the middle line. This appears objectionable, in as much as it would cause much more deformity of the nose. Mr. Liston carries the incision always around the ala of the nose, and thus this organ retains its natural appearance.]—*Id. from Hamburg Zeitschrift f. d. g. Med.*

---

*New Mode of tying Polypi of the Pharynx.* By Dr. Felix Hatin.—The tumour in this case was fixed by a large pedicle to the base of the cranium, and descended vertically into the pharynx, which it filled, reaching to the uvula; the soft palate was pressed downwards and forwards by it; the passage of the breath through the nostril was entirely impeded; the voice was what is improperly called nasal, and deglutition difficult. The operation was performed with a quickness and facility which M. Lisfranc admitted was surprising; and Dr. Hatin thus explained the method he adopted: In the ordinary method, an elastic sound must first be passed through one of the nostrils, and the posterior extremity, being brought forward into the mouth, is to be attached to the two ends of a ligature, in which a noose has previously been made; the sound is then to be drawn back, and the extremities of the ligature pulled into the nostril, while the noose passes towards the back of the mouth. The great difficulty is to preserve the loop of the proper dimensions, and to get it over the extremity of the tumour; a thing by no means easily accomplished, in consequence of the depth of the situation in which the polypus is fixed, and the convulsive movements caused by the fingers of the operator in the throat of the patient.

To overcome these difficulties, Dr. Hatin has invented an instrument (called *porte-ligature pharyngien,*) the construction of which is very simple. It consists of a flat metallic blade, curved at one extremity, which is to be introduced into the mouth through the noose in the ligature, and placed under the polypus, so that the ligature may be conducted over it. For the purpose of widening the curved extremity, so as to adapt it to the width of the tumour, two other narrow and flat metallic blades are fixed on the upper surface of the instrument, each of which is divided by a hinge. At the straight extremity of the instrument which is taken hold of, these two pieces diverge from each other, and are connected by a screw: when this is turned, the blades at the proximal end are brought nearer together, and diverge at the curved end, (beneath the polypus,) which is thus widened. To render the instrument complete, a stilette fixed to a spring, with two little hooks at its extremity, is placed in a groove at the lower side of the instrument; the ligature is fixed to the hooks, and, by pressing on a button at the end of the stilette, the noose is conducted at once to the end of the tumour, over which, by pulling the threads through the noose it may easily be made to slip. It is difficult to explain the mechanism of the instrument by a description; but the above will serve to give some idea of a contrivance by which a most difficult operatiom is rendered perfectly easy.

[From the difficulties attending its application, the ligature is hardly ever applied to the removal of polypi in the nose or fauces in this country. The instrument of Dr. Hatin seems capable of removing this difficulty in a great measure in tumours in the latter situation; but still one imperfect part of the operation must be the difficulty of drawing the noose tight round the base of the tumour, after getting it over the extremity: this can be but imperfectly accomplished by pulling the extremities of the ligature through the nose.] *Id. from Revue Méd. fran. et étran.*

---

*Case of Suspected Infanticide.* By Dr. Graff, of Darmstadt.—[A child having been found dead under suspicious circumstances, a medical inspection was directed to be made to determine the cause of death.]

*Externally*, the following appearances were met with. The skin was soft and shrunken, the cuticle easily peeling off. The countenance was wrinkled, and presented an aged appearance. About the neck there were marks of lividity, afterwards found to correspond with deep discoloration of the cervical muscles. On many parts of the head, especially posteriorly, there were patches from which the cuticle had been separated. There were no external wounds, with the exception of a small puncture on the outer side of the left foot. This wound contained a little blood, and its margin was somewhat livid and discolored; but there was no mark of swelling or inflammation in the surrounding structures. The nails of the fingers and toes were not fully developed. The child was of the male sex, but there was no appearance of testicles. The body weighed three pounds. The umbilical cord, about two feet in length, which had been completely torn from the placenta, lay on the left side of the body, and was partially twisted round the under part of the left foot.

*Internally.* On reflecting back the skin of the cranium some blood was found effused: and in the left parietal bone there was a fine fissure about an inch in length, extending to the sagittal suture. Blood escaped through this fissure from the cavity of the cranium. The brain was throughout highly congested: beneath the fissure in the parietal bone there was about a teaspoonful of thick black blood. The muscles of the neck were of a very dark colour. In the chest the diaphragm were found, much protruded upwards, the lungs of a dark colour and situated quite posteriorly.

Some blood was found extravasated in this cavity. The lungs with the heart and thymus attached readily sank in a vessel of cold water. The lungs separated from the other organs also sank: when divided, they were very firm, not crepitating, and the divided portions sank when placed in water. The foramen ovale was open. No particular appearances were met with in the abdomen. The stomach was small, and contained a viscid bloody fluid. The bladder was empty, and the large intestines contained meconium.

From this examination the following opinion was given:

1. *The child was not mature, being about from four to six weeks under the full period.* The proofs of this were: the length, the weight, the shrunken appearance of the body, especially the limbs; the aged appearance of the face; easy separation of the cuticle; the great elasticity of the bones of the cranium; the cartilaginous state of the ribs, and imperfect development of the nails.

2. *The child had either not breathed or breathed but imperfectly.* This was established by the situation, colour, and consistency of the lungs; their sinking in water entire and divided; absence of crepitation; the small quantity of blood contained in them, and the open foramen ovale.

3. *Nevertheless, the child was born alive.* The evidence in favour of this was: unusual lividity of the muscles of the neck indicating compression at that part, and confirming the statements of those who found the child with a cloth tied tightly round its neck; the great ecchymosis about the scalp, with the fissure in the parietal bone and extravasation of blood beneath. Some rare cases are on record, in which under difficult labour the cranial bones of the child have become fractured by the muscular contraction of the uterus alone; but the occurrence of such an accident is altogether improbable, if not impossible, in cases where, like the present, the child is immature, the cranial bones elastic, and the sutures yielding. If the fracture and extravasation occurred after birth, it follows that the child must have been living; for otherwise it is impossible to admit that such an extravasation could have taken place. Another proof of live birth exists in the fact of blood having been found effused in the chest. In some instances, twisting of the umbilical cord around the neck may lead to asphyxia; but this view was here inadmissible, because, when the body of the child was found, instead of the umbilical cord *a cloth was tied fast around the neck.*

Allowing that the preceding facts show the child had lived after birth, the supposition of suffocation during birth through the twisting of the umbilical cord, cannot of course be entertained. If any cause operating after birth had produced the effusion in the chest, it is impossible that the child should have been born dead.

4. *The death of the child was most probably due to strangulation,* through the cloth found on its neck, or simultaneously through the hinderance to respiration, and the injury to its head. This opinion is borne out by the apoplectic state of the vessels of the head, the large extravasation in the chest and head, the wound in the left foot, which could not have been produced in the womb or during birth; and which on account of the ecchymosis accompanying it, must have occurred during life.

[REMARKS. This case offers some important reflections in relation to the proofs of infanticide. We fully agree with the reporter in the first two conclusions; viz. the immaturity of the child, and the fact of its not having breathed or breathed but imperfectly: but we do not see that the *medical* data which he had to guide him, justified so exclusively an opinion of this child having necessarily lived after birth. Lividity and ecchymosis about the neck and scalp offer no evidence of live birth; for experience shows that these may be equally produced during birth, and the child not come into the world alive. The fissure in the parietal bone is assumed to have been indicative of violence, merely because the bones were elastic and yielding; but, from the description of it, we are inclined to believe that it was probably a fracture from the efforts of the uterus during birth. Murderous violence to the head after birth is rarely confined to the production of a *slight fracture* in one parietal bone. At any rate, in the absence of direct evidence as as to the origin of so slight an injury, the presumption is as strong for natural as for violent causes. The reporter seems to imagine that extravasation of blood can only take place in the *living* child; but it is well known that precisely similar appearances may result from violence to the body of one which is recently dead. The presence of extravasation or lividity cannot therefore be taken in an abstract view, as any evidence of live birth. The injury on the foot bore more clearly the marks of design; and this, together with the violence about the head, seems to show, that the whole of the body of the child was in the world when it was produced; but whether the child was actually living or recently dead at the time, it does not establish. Perhaps the best evidence of this child having been living and wilfully destroyed, we have in the circumstances, especially in the fact that a cloth was tied tightly round the neck; for why should this be done to a child already dead? At the same time, this murderous violence might have been used towards it *during the act of birth.* The non-inflation of the lungs is of course no objection to the child having come into the world alive, and lived long enough to be the subject of murder.]—*Id. from Henke's Zeitschrift.* 1838.

---

*Case of Suicidal Strangulation.* By Dr. CARGANICO, of Darkehmen.—A peasant was found lying dead close to a well-frequented road and in the neighbourhood of dwelling-houses. The following facts came to light from the judicial and medical examination.

The body was lying stretched out at full length on the abdomen, the arms placed at the sides, and the hands half-closed. There was no mark of violence on the person or dress, or appearance of struggling or trampling on the grass around. The deceased appeared to be a robust man of about fifty; the countenance was pale, with the exception of a slight cadaverous ecchymosis about the root of the nose. The eyes and mouth were naturally closed, the lips pale, the tongue in its natural position not swollen, the ears not discoloured, the features having a very placid expression. The hands which were half-closed, as is usual in death, presented no traces of violence. Particular attention was now paid to the neck: the cravat worn by the deceased was found carried twice round the neck in the customary way, and tied by a knot in front. On the left side a small stick about four inches and a half long had been thrust between the two folds of the cravat, then twisted by a half-turn, and so brought round that the lower end rested firmly against the angle of the jaw, which prevented it from returning. This had so compressed the neck by tightening the cravat, that it was impossible to introduce a finger under it. Near the body a branch of a tree was found, from which the stick had evidently been cut.

The neck between the cravat was scarcely depressed, and the skin was free from all sugillation or desiccation. The larynx, trachea, and os hyoides were normal. The body was free from all traces of violence, and there was not even the least sign of cadaverous lividity. Being satisfied from these facts that the deceased had destroyed himself by strangulation, the reporter did not make an internal inspection, but proceeded to draw up a medical opinion. He observes that a case of such interest was deserving of closer examination, in which we fully agree with him; but, at the same time, he assigns no satisfactory reason for having omitted to institute it.

1. The *cause of death* was apoplexy, not asphyxia, i. e. the ligature did not act by interrupting respiration, but by compressing the cervical vessels and preventing the free circulation of blood through the brain. The proofs of this were: paleness of the countenance and lips, absence of lividity in the body. The want of a well defined and ecchymosed depression in the course of the ligature is an additional proof that asphyxia was not the cause; while in apoplexy from strangulation this ecchymosed depression is often wanting. The absence of a mark he satisfactorily accounts for, by the fact that the compressing material was soft and wide, and that it was not very tightly applied, although still sufficiently so to effect the cerebral circulation.

2. *Was the strangulation suicidal?* This was rendered in the highest degree probable, if not certain. Suicidal strangulation is not very common, but in this case the means employed for accomplishing it, as well as the whole of the circumstances, were only intelligible upon such a presumption. Strong corroborative proofs existed in the absence of violence to the person and dress, the position of the body, and placidity of the countenance. A healthy and strong man like the deceased could not have been easily destroyed by others in the manner described, without indubitable evidence of the fact being left on his person. Again, the ligature to the neck had not been violently applied as it would have been by a murderer, but the compression of this part had been gradual and comparatively slight. Murder by strangulation would have been indicated by extensive injury to the skin, and probably to the deep-seated organs of the neck, at least this is what is commonly observed: but none of these signs existed in the case of the deceased.

[REMARKS. Two facts are we think pretty clear in this case, and they are fortunately all that the law requires to be established: 1, that the deceased died from strangulation; 2, that he strangled himself. Whether the ligature operated by impeding the cerebral circulation, by preventing respiration, or in both ways, is a point of no importance in a judicial light, and highly absurd to inquire into as a medical fact, where the *exterior of the body* only has been seen. Casper's observations, already reported in this Journal, establish that the production or non-production of an ecchymosed mark by a ligature in strangulation, can furnish no evidence as to whether death took place by apoplexy or asphyxia; and we think it would have been somewhat surprising to have met with ecchymosis under the circumstances of the present case. The reporter first assumes that the deceased died from apoplexy, next that the suicides who hang themselves die more frequently from this cause, and then he comes to the conclusion that the deceased's having died of apoplexy is a proof of his having committed suicide! There is fortunately good evidence of suicide in the facts themselves, thus rendering it unnecessary to resort to such weak hypothetical reasoning as this.]—*Id. Ibid.*

---

*On the Structure and Functions of the Cerebral Nerves of the Frog.* By Professor VOLKMANN, of Dorpat.—[This article notices not only the origin and functions of the cerebral nerves, but describes likewise in great detail their course, and the muscles which they supply. Our extracts shall be confined to the first two heads.]

1. *On the Origin of the Nerves.* Only eight separate nerves arise from the brain of the frog; as the facial, glossopharyngeal, accessory, and hypoglossus are supplied by branches from other nerves. The facial is a branch of the auditory; the ninth and eleventh pairs are contained in the tenth; and the first cervical nerve supplies the place of the hypoglossus. The motor oculi arises from the crus cerebri behind the tuber cinereum to which the pituitary gland is attached. The nerve runs outwards and forwards, and passes behind the optic nerve through a cartilaginous plate, which represents the great wing of the sphenoid bone. The pathetic arises from the posterior and upper border of the corpora quadrigemina, passes outwards and downwards, and traverses the cartilaginous plate of the sphenoid, a little way above the foramen of the motor oculi. The trigeminus arises from the external border of the medulla oblongata,—it runs forwards and outwards, and traverses the bony portion of the sphenoid, forming, in the foramen through which it passes, a reddish ganglion, which receives several nerves. The abducens takes its origin from the anterior fissure of the medulla oblongata, and ends in the ganglion of the trigeminus. The auditory nerve arises immediately posterior to the abducens, and soon divides into two branches, one of which passes into the ganglion of the fifth. The pneumogastric nerve arises from the outer edge of the medulla oblongata, posterior to the auditory nerve, and receives some nervous fibres which appear to represent the glossopharyngeal. It is not joined by any fibres which can be supposed to represent the accessory of Willis.

II. *On the Functions of the Nerves.* The motor oculi, judging from its course, is a mixed nerve, and endows the muscles which it supplies alike with motion and sensation. It is distributed to all the muscles of the eye except two, and as a voluntary muscle cannot be supposed to be without sensation, seeing that this quality regulates the degree and direction of the motion, it must in consequence be admitted that the motor nerve of the muscles of the eyeball contains also sensory fibres. On irritating the motor oculi of a recently killed frog, within the cavity of the skull a variety of motions were induced; the eyeball was turned in various directions, and rolled as if by the action of the inferior oblique. On preparing the parts so as to bring several of the muscles into view, and again irritating the nerve, spasmodic twitches were observed in several of the recti, but no effect was produced in the superior oblique, in the external rectus, or in the suspensorious muscle.

The pathetic nerve of the frog, regarded in an anatomical point of view, must be considered as purely motor. On irritating the root of the nerve a motion of the eyeball is produced, which, from its nature, must be dependent on the action of the superior oblique; and when the parts are prepared so as to show the muscles, and the nerve is then irritated, the convulsions are seen confined to that muscle. Division of the pathetic, before it unites with the nasal nerve, does not seem to cause the animal pain, but it should be remembered that frogs when tormented occasionally suppress the expression of pain. It is more difficult to determine by experiment the function of the sixth pair, as its fibres traverse the ganglion of the trigeminus. On irritating its root the eyeball was drawn violently inwards, and was covered by the membrana nictitans, or in some instances it was turned backwards. On displaying the muscles of the eye and then applying irritation to the nerve, contraction of the fibres of the rectus externus and suspensorious was distinctly observed; and on dividing, in another frog, the third and fourth pairs, and irritating the medulla oblongata with a needle, the eyes were violently drawn back into their sockets, and covered by the membrana nictitans. They remained shut a considerable time, and then suddenly opened, the eyeballs being drawn forwards, and the membrana nictitans downwards. The irritation reapplied reproduced the above phenomena. In another frog the sixth pair of both sides was divided, and the motor oculi and the pathetic were then irritated. The eyeballs were in consequence slightly moved, but they were never withdrawn into their sockets, nor were they covered by the membrana nictitans. Dr. Volkmann therefore considers the abducens as the most important motor nerve of the eyes; it is much thinner than the motor oculi, but this depends upon the latter being a mixed nerve and including sensory fibres, whilst the former is purely motor.

The trigeminus is a mixed nerve, and supplies both integument and muscle. Irritation of its root produces contraction of the mental and temporal, of the mylohyoid and nasal muscles, but no motion of the muscles of the eyeball.

On irritating the facial nerve within the cavity of the skull, convulsions of the vertebro-maxillaris and tympano-maxillaris were produced, and on applying the stimulus of galvanism, not only these muscles were affected, but also the stylohyoideus anterior, and in the male the muscular sac of the larynx, or in the female the slender muscular fibres of the pharynx.

The nervus vagus is a mixed nerve, formed from the union of the ninth, tenth, and eleventh pairs. Irritation of its root within the cavity of the skull produced convulsions in the levator scapulæ inferioris, the stylohyoideus posterior, the stylopharyngeus, and the muscles of the larynx. The glossopharyngeal branch was divided, but irritation of its extremity produced no convulsions in the muscles which it supplies; it is therefore considered as purely a sensory nerve. The ramus recurrens is a motor branch; irritation of it caused convulsions in various parts of the larynx; in particular the glottis was drawn to the side of the irritated nerve, and would unquestionably have opened, had the opposite side been fixed and not yielded mechanically to the contraction of the irritated muscles. The influence of the vagus upon the motions of the heart is very peculiar. The brain and spinal cord of a frog were destroyed, and the anterior extremities and sternum carefully removed, so as to expose the trunk of the nerve. About a quarter of an hour after the death of the animal, galvanic stimulus was applied to the vagus by means of eight pairs of four inch square plates, at the same time constantly breaking and reconnecting the chain. Immediately before the experiment, the heart was beating thirty strokes a minute; in the second minute after application of the stimulus, it beat thirty-three strokes, and continued to do so during the third, fourth, and fifth minutes, when the experiment was interrupted. Three quarters of an hour after death the heart was beating twenty-nine strokes a minute; the galvanic stimulus was reapplied, and in the second minute afterwards it beat only eleven strokes, in the third thirty-one, and in the fourth thirty-four. The small number of strokes during the second minute appeared to be owing to intermission. The experiment was repeated two hours after death, when the heart was beating twenty-nine strokes in the minute. During the second minute after application of the galvanism the number of beats was twenty-six, and during the third only sixteen. The difference was owing to intermission; the strokes followed at regular intervals till an intermission of perhaps nearly half a minute occurred, and then they again followed in regular succession. Irritation of the vagus likewise augmented the peristaltic motions of the stomach and bowels. The first cervical nerve is also a mixed nerve, and irritation of its root produces convulsions in the muscles which it supplies.

[The above sketch is necessarily very imperfect, as we have been obliged to omit many anatomical details, the knowledge of which would have removed several obscurities. For their solution we must refer the reader to the original paper.]—*Ib.*, *from Müller's Archiv*, 1838, Heft i.

---

*Medical School, Berlin.*—With the exception of Paris, there is no medical school on the continent which enjoys so high a reputation as that of Berlin, or which ranks among its professors so many distinguished men. The great superiority of the professors over those in other German Universities must be principally ascribed to the fact of their generally enjoying extensive practice, and being men of experience—not such as German professors usually are, men who read and write on disease, but have little practical knowledge of it. It must also be, in a considerable degree, attributed to the zeal of government, in using every opportunity of attracting eminent talent to the metropolis.

There are, in Berlin, two very large hospitals, the Old and the New Charité; also a smaller institution, called the Universität's Clinicum; and a Lying-in Hospital. These are the chief medical institutions; and all the most important cliniques are held in the Old Charité, which is also much the greatest establishment. At the head of the cliniques must be placed that of the veteran President Rust. The mode in which it is conducted is the following:—A patient is brought in, usually in his bed, into the centre of the operating theatre. One or two students are then called on by name, and required to discover the nature of the disease, and to propose a mode of treatment. The professor cross-questions the students on the case, and then proceeds to explain his own views regarding it. After this the patient is removed back to his ward, and it is well if the pupils can afterwards obtain any knowledge of the further progress of the case. For several years Professor Rust has not operated, and accordingly the operations in his clinique are performed by his colleague, Professor Dieffenbach, famed for his rhinoplastic dexterity. There is also a smaller surgical clinique, where the pupils are allowed to perform the minor operations, under the superintendence of Professor Von Gräfe. He, however, as well as Professor Dieffenbach, are esteemed more as operating than as general surgeons.

There are two medical clinques,* the most important that of Dr. Wolff; the other, in which Latin is spoken, is temporarily conducted by Professor Wagner. These are the only two cliniques which correspond, in their arrangements, to the wards of English hospitals, *i. e.* which are visited every day by the physician and pupils, and where the progress of a case can be watched and studied. In them the cases are all treated by the pupils under the direction of the physicians. From the small number of cases, and from the crowd of students usually present, it is difficult to learn much. The clinique of Dr. Barez for the diseases of children, is undoubtedly one of the very best in Berlin; it is well worthy of attention, as no similar institution, I believe, exists in Germany; for the diseases of children are too little studied abroad as well as at home. The arrangements correspond nearly to those of an English dispensary, but the most interesting cases are selected, and form the subjects of clinical remarks. There are one or two small wards for in-patients, holding about thirty beds; but the out-patients who form the great mass, are visited in their houses by the students. Here, as indeed very generally in German practice, the oil of the cod's liver is employed with the most marked success in cases of scrofula, and it is surely deserving of a fair trial in this country.

Equally excellent is the eye clinique of Professor Jüngken. Here, also, the cases are chiefly those of out-patients, there being only two small wards which are chiefly set apart for the cases that have been operated on. The accuracy of diagnosis, and the great excellence of the clinical instructions of Professor Jüngken, are universally acknowledged. The syphilis in wards, where mercury is not used in any form, is under the direction of Professor Kluge. This clinique affords great advantages to those who are anxious to study the various forms of venereal disease. There are two obstetrical cliniques, one in the hands of Professor Kluge, the other in those of Professor Busch. There is also a clinique for for cases of insanity, of which Dr. Ideler is physician.

The medical lectures are generally delivered within the University, although some (as those of Professor Mitscherlich) are given in private houses. The anatomonical theatre and dissecting-rooms are in a separate building, at some distance from the University, and by no means remarkable for the excellence of their arrangements. The celebrated Professor Müller lectures in winter on anatomy, and in summer on physiology; and here it may not be out of place to remark, that attendance on the whole course of anatomy costs two louis-d'ors, while, for as much more, he is supplied with abundance of subjects for dissection; the whole course, with dissections, thus costing 3*l.* 8*s.* Notwithstanding the excellence of his lectures, it is to be regretted that Professor Müller does not use the aid of dia-

* The best medical clinique in Germany is probably that of Professor Krunkenberg, in Halle.

grams, which are of great assistance, especially to beginners.

On materia medica, the lectures are those of the famous botanist Sink, of Osann, of Schultz, and of Mitscherlich*, brother of the chemist.

Almost all the lecturers on the different branches of medicine are hospital physicians, and it is unnecessary to particularize them; but perhaps the lectures of Professor Romberg deserve special notice. Among the private courses, or "*privatissima*," as they are termed, there are three which deserve the attention of every stranger who makes a stay in Berlin; and in no other place, probably, are courses of the same kind and of equal excellence to be found. They are Jüngken's eye, and Dieffenbach's plastic operations, and Schlemm's course of all the common operations of surgery. With lectures on more general subjects, Berlin is very well supplied. Mitscherlich and Rose lecture on chemistry, Sink and Kemth on botany, Ehrenberg on the physiology of the infusoria, Rose and Weiss on mineralogy, and Von Dechen on geology.

The German system of clinical instruction appears to every stranger to be very good, so far as it goes, and affords many advantages to the beginner, but by the more advanced student the want of real hospital practice is severely felt;† nevertheless, it admits of little doubt, that our medical schools might be improved by the partial introduction of the German system.

For Germans, it is necessary to have studied in a gymnasium until they are declared ripe for the university, and then to have studied medicine in a university for four years, in order to obtain the degree of M. D. This degree is nowhere held in high repute, and is chiefly viewed as a preliminary to the "staats examen," which must be undergone before a license to practise can be obtained in Prussia, or in most other states. This last examination is usually extended through several months. The candidate is first examined in anatomy, and required to make a preparation of some part of the body; he is next examined in surgery, and is called on to perform some operations on the dead body. After separate examinations in the different branches of medicine, he has to treat the case of an out and of an in-patient at the hospital, and it is only after passing through these ordeals that he is allowed to practise. The stranger will not find the same liberality in admission to the cliniques of Berlin which he meets with in London; and it is often necessary for him to ask the leave of a professor, before he can be admitted to a particular ward.

It might be a curious inquiry to ascertain what are the causes of the present state of medical literature in Germany: why there is such a want of good works on medicine and surgery, along with such an abundance of excellent books on anatomy, physiology, and materia medica. Three causes suggest themselves. 1st, the theoretical and generally unpractical turn of the German mind; 2d, the want of public hospitals managed as in England; and 3d, what appears to be far the most important cause, the mode of appointment of professors in the German Universities. The aspirant to a professorship commences his career by obtaining leave to lecture under the name of "*privatim docens.*" His object is to obtain some name as soon as possible, in order to secure early promotion to a professorship. This he can most readily do by writing something; and, whether the individual has had experience in his profession or not, he writes his book. In anatomy and physiology, where every one has the means of making new observations, this is very well; but in medicine and surgery it has the most prejudicial effects. The same evil continues after the *privatim docens* has attained the rank of professor, when it is a matter of profit to him to have his system of medicine as a handbook for his class. The general result of this is, that for practical improvements in medicine Germany has always to look to England and France, although it is not meant to be denied that from time to time works of great practical value appear. English books on medicine are much read in Germany, and all books, whether good or worthless, are immediately on their appearance translated into German, and you see Johnson's Course of Human Life, and Curtis on the Ear, on the same table with Stokes on the Chest, or Copland's Dictionary.

Among the theories influencing German practice, perhaps none has so much weight as one which has of late years come much into fashion, and which attributes the origin of most diseases to be in the abdomen. By this phrase are meant all the abdominal affections to which many of middle age are especially disposed. The German professor examines the internal surface of the under eyelid, sees that the vessels have a congested and venous aspect, and immediately exclaims that the cause is some abdominal obstruction. This doctrine seems to be taking the place of that which attributed so much to suppressed secretion, and to checking the perspiration of the feet in particular.—*London Medical Gazette.*

---

*A case of Fracture of the Coracoid Process of the Scapula, with partial Dislocation of the Humerus forwards, and Fracture of the Acromion process and of the Clavicle.* By John F. South.—The author was induced to lay this case before the Society, as from being verified by dissection, it is adequate to remove those doubts which have been often entertained of the occurrence of such an accident as fracture of the coracoid process. The author saw the patient about an hour after he had fallen from a scaffold thirty feet high. In addition to the injury of the shoulder, he had a wound over the coronal suture, with surrounding contusion, but no evidence of fracture, or injury to the brain, though blood streamed freely from the left ear.

* Dr. Mitscherlich has lately commenced the publication of a System of Materia Medica, likely to prove of great value.

† There seems to be no good reason why this great defect might not be remedied, by throwing open the greater part of the wards of the Old Charité to the students.

There was also extensive injury to the elbow joint, with fracture of the olecranon. From a careful examination of the shoulder-joint, the author was led to the conclusion that the humerus was dislocated under the clavicle of the same kind, though not to the same extent as the so-called dislocation under the pectoral muscle. The dislocated head of the humerus was replaced by lifting the neck outwards with the thumb, and rotating the arm, and its replacement was indicated by a grating sound. After four days, the patient having died from his several injuries, the author had an opportunity of examining the shoulder, of the appearances of which he gives the following account:—"On turning back the integuments, a small quantity of effused blood was found on the front of the shoulder; and, to my surprise, a fracture of the clavicle about a third of its length from the acromial extremity, with, however, but little displacement."

"The acromion was broken at the usual place, about an inch from its extremity, but not at all displaced, as the periosteum had not been lacerated. The coracoid process was found broken about half an inch from its tip into two unequal pieces, the smaller of which remained connected above, with a piece of the triangular ligament still attached to the acromion, and below to the short head of the biceps muscle, which had pulled it down as far as the ligament would allow. This muscle was torn from the coraco-brachialis about an inch, and to the top of the conjoined tendon of the latter, and of the lesser pectoral muscle, was attached the larger portion of the broken coracoid process," &c.

The author, in conclusion, makes some observations on the partial dislocation of the humerus, which he conceives can only take place in conjunction with fractures of the coracoid process.

*Ibid.*

---

*Anatomical Researches into the Comparative Structure of the Cutaneous and Mucous Membrane.* By M. Flourens.—In the skin of the European, the dermis is covered by two layers of epidermis, the one internal and the other external; in the black or coloured races there is, beneath these two membranes, an organ for the secretion of colouring matter. In the tongue, whether of man or of quadrupeds, there exists between the dermis and epidermis a peculiar body, called mucous body (corpus mucosum.) This body, which appeared to Malpighi, who obtained it by boiling, to be reticulated, is proved by the more exact process of maceration, to be continuous and membranous; of the two layers of epidermis in the skin of the European, the internal is the most coloured; in the tongue the mucous body is the seat of all partial discolorations. The mamma, in the human species, is surrounded by an areola or circle of a lighter or darker brown, or bister-colour. This colour is proved by careful maceration to be due to the internal epidermis, which is of a deep brown; but when seen through the external layer has a light grayish aspect. In the skin, then, of the European, the second or internal epidermis is the seat of colour. In every part of the body this membrane is more coloured than the external layer; it is the seat, as we have seen, of the discoloration of the nipple; it is upon this, too, that the sun produces its tanning effect. The dermis is the seat only of freckles, and other minute discolorations. The tongue may be taken as the type of an entire group of mucous membranes; externally it is covered by the epidermis, beneath the epidermis lies the mucous body, and beneath this again the dermis with its papillæ. The mucous body which exists in the tongue, and which is found also on the palate, cheeks, and throughout the entire cavity of the mouth, extends still farther throughout the œsophagus. At the point where the œsophagus terminates and the stomach begins, an entirely different structure, which will form a subject for future inquiry, commences.

The characters of the mucous body are everywhere the same. In man it is white, in the ox it is the seat of those partial discolorations which are so often seen upon the palate and tongue of that animal. Its tissue is peculiar; it is rendered more compact, and, when its colour is white, becomes more white by boiling. It consists of layers superimposed and adherent. The second or internal epidermis is very thin and fine; it is covered in the areola of the nipple by a more or less distinct layer of pigmentum, and readily passes to a diffluent state. There is no doubt that the fable of a mucous membrane belonging to the skin took its rise from this state of the internal epidermis. This membrane can be obtained only by a certain degree of maceration; if the process is not carried on long enough it is detached with the external epidermis; if it is pushed too far, the membrane is dissolved away. Between these two degrees of maceration there is a point at which the internal epidermis is separated as a continuous and distinct layer. Those anatomists who have not continued the maceration long enough have denied the existence of a mucous membrane; those who have pushed the process too far have spoken of a mucous body, of a species of mucosity, or of a mucous and gelatinous liquid, (Meckel.) Maceration, properly conducted, demonstrates, in place of this mucosity, a *bonâ fide* membrane, thin, and coloured—the internal epidermis.

The internal epidermis and the mucous body, then, are two distinct tissues, the former being to the skin what the latter is to the group of mucous membranes of which we are speaking. It is important to determine the precise point at which the one terminates and the other begins. If we examine the lips carefully, we trace a distinct continuity, on the one hand, between the external epidermis of the skin and the epidermis of the mucous membrane; on the other, between the dermis of the skin and that of the mucous membrane; but at the line which separates the pale from the coloured part of the lip, the mucous body takes the place of the internal epidermis.

The mucous membrane which covers the tongue, extends, in the ox, over the entire cavity of the mouth, throughout the œsophagus, and over the three first stomachs; in the horse, it terminates in the stomach itself. The structure of this membrane varies somewhat in the several parts which it covers. It is thinner on the cheeks than on the tongue. Near the lips it is furnished with numerous long papillæ. Each of these papillæ is surrounded by two sheaths, the one furnished by the epidermis, the other by the mucous body. The same structure prevails in the palate, the dermis of which is arranged in transverse lines bristling with papillæ. The mucous body, which is the seat of all discolorations, is composed of layers superimposed and adherent, and these layers again consist of perpendicular *blades*. The mucous membrane of the œsophagus and of the three first stomachs of the ox closely resembles that of the tongue and mouth. The papillæ, though differing in shape and arrangement in the several parts, are everywhere covered by two sheaths derived from the mucous body and from the epidermis. After sufficient maceration these sheaths may be detached from the papillæ like the fingers of a glove. The mucous membrane of the horse differs but little from that of the ox. On the palate we observe the same transverse lines, but without papillæ. It covers a part of the stomach, and exactly resembles the lining membrane of the three first stomachs of the ruminantia.

In the horse, then, as well as in the ox, there exists an entire group of mucous membranes precisely similar to the membrane covering the tongue. In the one, as in the other, this membrane lines the entire cavity of the mouth. In the horse, it extends also through the œsophagus and first part of the stomach,—in the ox, through the œsophagus and three first stomachs.—*Brit. and For. Med. Rev., from Gaz. Méd. de Paris. Mars*, 1838.

---

*Case of Congenital Transposition of the Viscera.* By Dr. Tonelli, of Rome.—A male infant, born at the full time, and in other respects properly developed, presented the following appearances when seen by the writer, on the third day from the birth. A tumour of oblong shape, resembling a moderate-sized cucumber, was attached to the umbilicus by a peduncle which spread out into a funnel-shaped base, about nine inches in circumference. The tumour itself extended longitudinally from the middle of the sternum halfway down the thighs. [Its longitudinal measurement is not more particularly stated.] It projected in front of the navel five or six inches. Its transverse diameter, opposite the navel, was about six fingers' breadth. The lower portion of its investing membrane was pellucid, yellowish, and evidently contained a fluid. The upper portion resembled the common integuments, and had a soft, elastic feel. The navel-string adhered to the right side of the tumour for the space of about four inches. The infant had properly voided its urine and meconium, and had begun to take the breast. It died shortly after this inspection. On the lower part of the cyst being punctured, there flowed out about six ounces of yellowish serum. When the upper part was laid open, all the viscera were found within, arranged in a relative position analagous to that which they would have naturally occupied within the abdomen, with the exception only of the kidneys, bladder, and rectum. On tracing the cardiac extremity of the stomach, it was found to bend upwards, and enter the umbilical opening by the side of the umbilical vessels. In like manner the extremity of the colon was seen to enter the abdomen. The cyst was now removed by cutting through the peduncle, the orifice of which measured two inches and a half in diameter. The abdominal cavity was found rather smaller, and the tendinous attachments of the diaphragm, posteriorly, somewhat lower than natural. The kidneys and urinary bladder were found in their natural positions, and the stomach and colon were found continuous with the œsophagus and rectum respectively, as was to be expected from the regular performance of the functions of those organs. Dr. Tonelli considers the present case as one not hitherto described. He refers to the case of thoracic hernia recorded by Mr. Morgan, of the Bristol Infirmary, and to the congenital transposition of the viscera in an adult observed at Calcutta, by Mr. Hardy, (see both articles in Medical Gazette, vol. xii. pp. 79 and 673,) but justly remarks that these cases bear no analogy with the present one.—*Ibid, from Annali Universali di Medicina.* Vol. lxxxii. *Aprile, Maggio, Giugno*, 1837.

---

*Application for Blistered Surfaces.*—Sir B. Brodie orders the following preparation where a blister becomes troublesome.

*Prepared Chalk ; Olive Oil*, of each five drachms;
*Rose water*, two ounces. Mix.

*Lancet.*

---

*Treatment of Cicatrices from Burns.* By Sir B. Brodie.—Sir B. B. follows the plan of treatment recommended and practised successfully by Mr. Cæsar Hawkins, in such cases;—soaking the cicatrix in neat's foot oil.—Sir Benjamin states that he has generally been unsuccessful with Mr. Earl's operation.—*Lancet.*

---

*Mr. Wakley's election to the office of Coroner.*—Mr. Wakley, long known as the Editor of the London Lancet, and as member of parliament from Finsbury, has recently been elected Coroner of Middlesex county, by an immense majority over his opponent Mr. Adey, the attorney candidate.

---

*Paris Edition of Palmer's Hunter.*—A translation of Mr. Palmer's complete edition of the works of the celebtated English Surgeon John Hunter has been made in Paris, and is about publishing there.

